# Glutamate Transporters in Hippocampal LTD/LTP: Not Just Prevention of Excitotoxicity

**DOI:** 10.3389/fncel.2019.00357

**Published:** 2019-08-06

**Authors:** Joana Gonçalves-Ribeiro, Carolina Campos Pina, Ana Maria Sebastião, Sandra Henriques Vaz

**Affiliations:** ^1^Instituto de Medicina Molecular João Lobo Antunes, Faculdade de Medicina, Universidade de Lisboa, Lisbon, Portugal; ^2^Instituto de Farmacologia e Neurociências, Faculdade de Medicina, Universidade de Lisboa, Lisbon, Portugal

**Keywords:** glutamate transporters, synaptic plasticity, tripartite synapse, astrocytes, NMDAR

## Abstract

Glutamate uptake is a process mediated by sodium-dependent glutamate transporters, preventing glutamate spillover from the synapse. Typically, astrocytes express higher amounts of glutamate transporters, thus being responsible for most of the glutamate uptake; nevertheless, neurons can also express these transporters, albeit in smaller concentrations. When not regulated, glutamate uptake can lead to neuronal death. Indeed, the majority of the studies regarding glutamate transporters have focused on excitotoxicity and the subsequent neuronal loss. However, later studies have found that glutamate uptake is not a static process, evincing a possible correlation between this phenomenon and the efficiency of synaptic transmission and plasticity. In this review, we will focus on the role of the increase in glutamate uptake that occurs during long-term potentiation (LTP) in the hippocampus, as well as on the impairment of long-term depression (LTD) under the same conditions. The mechanism underpinning the modulatory effect of glutamate transporters over synaptic plasticity still remains unascertained; yet, it appears to have a more prominent effect over the *N*-methyl-D-aspartate receptor (NMDAR), despite changes in other glutamate receptors may also occur.

## Introduction

Glutamate was first classified as a neurotransmitter in the 1950s (Hayashi, 1952), and is presently acknowledged as the major excitatory neurotransmitter in the mammalian brain. Having a pivotal role in neuronal signaling, it has been vastly implied in several brain functions, such as cognition, memory, and learning (for review see Zhou and Danbolt, 2014). Within the Central Nervous System (CNS), glutamate acts by binding to receptors coupled to ionotropic channels, as *N*-methyl-D-aspartate (NMDAR), α-amino-3-hydroxy-5-methyl-4-isoxazole propionic acid (AMPAR) and kainite receptors, and metabotropic glutamate receptors (mGluRs). mGluRs can be subclassified into three distinct categories in accordance to sequence homology, G-protein coupling and ligand selectivity: Group I includes mGluR1 and mGluR5, Group II includes mGluR2 and mGluR3, and Group III includes mGluRs 4, 6, 7, and 8 (for review see Niswender and Conn, 2010). Paradoxically, in spite of its critical role in overall CNS functionality, glutamate can also act as a neurotoxin (Choi et al., 1987). When in abnormally high concentrations, glutamate can severely damage neurons, or even lead to neural death, by overactivation of NMDA or AMPA receptors in a process referred to as excitotoxicity, suggesting a thorough regulation of its concentration is required for proper neuronal signaling. This regulation is primarily performed by high-affinity glutamate transporters, but also by passive diffusion, albeit to a lesser extent (Barbour and Häusser, 1997).

Neurotransmitter uptake is crucial for normal synaptic transmission, being performed by astrocytes and neurons. For this purpose, both cell types express distinct transporters, each corresponding to specific neurotransmitter, which are responsible for the recycling and the regulation of the synaptic concentration of the neurotransmitter, directly influencing several aspects of synaptic communication, such as the duration of postsynaptic responses. Accordingly, glutamate transporters are responsible for clearing glutamate from the extracellular space into the cell, in order for it to be either metabolized or recycled, in compliance with the cell’s needs. So far, five high-affinity glutamate transporters have been identified and characterized in the CNS: Glutamate Aspartate Transporter (GLAST) (Storck et al., 1992), Glutamate Transporter type 1 (GLT-1) (Pines et al., 1992), Excitatory amino-acid transporter 4 (EAAT4) (Fairman et al., 1995) and Excitatory amino-acid transporter 5 (EAAT5) (Arriza et al., 1997).

Several studies support the notion that glutamate transporter expression is not uniform among distinct cell types and brain regions. GLT-1 and GLAST are the most copious transporters in the forebrain (Danbolt, 2001; Takahashi et al., 2015), being responsible for approximately 90% of total glutamate transport and subsequently, for maintaining extracellular glutamate at optimal levels, thus preventing excitotoxic events. This is further supported by studies of GLT-1-KO (Tanaka et al., 1997) and GLAST-KO (Watase et al., 1998) phenotypes, as mice suffered from lethal seizures and impaired motor coordination, respectively, strongly indicating glutamate transporters are key regulators of neuronal excitability. Immunostaining for GLT-1 in tissue reports a more prominent expression in the cortex and hippocampus (Danbolt, 2001), being found almost exclusively in glial cells. This transporter has three known isoforms (GLT-1a, GLT-1b and GLT-1c) with different relative expressions. EAAC1 (EAAT3) is exclusively expressed in neurons. GLAST expression is more pronounced in the cerebellum, EAAT4 in the cerebellar Purkinje cells and EAAT5 in the retina (Danbolt, 2001).

Glutamate transport mediated by high-affinity transporters in an electrogenic process characterized by the translocation of net positive charge during each transport cycle. The inward transport of a glutamate anion is coupled with three Na^+^  ions, accompanied by the simultaneous outflux one K^+^ ion, indicating glutamate transport is a sodium-dependent process driven by electrochemical gradients across the cell membrane (Kanner, 2006).

As aforementioned, glutamate transporters prevent excitotoxicity, a phenomenon vastly implied in multiple neurological disorders, such as epilepsy, Parkinson’s disease (Van Laar et al., 2015), Alzheimer’s disease (Hynd et al., 2004; Esposito et al., 2013), and Amyotrophic Lateral Sclerosis (ALS) (Van Den Bosch et al., 2006), thus emphasizing the importance of a thorough regulation of glutamate uptake. Indeed, the majority of studies regarding glutamate uptake have focused on its impact on neuropathologies, however, in more recent years, there has been a gradually increasing interest in the putative role of these transporter in synaptic transmission. Although glutamate transporters do not exactly bind to glutamate, they do compete with glutamate receptors for this neurotransmitter, implying receptor activation can be modulated by transporter activity. Since the activation of postsynaptic glutamate receptors affects synaptic transmission, glutamate transporters are able to influence synaptic transmission via a regulation of glutamate levels. Modulation of glutamate transport can be associated with a variety of factors ranging from neural stimulation to protein synthesis and ion channel, as will be further discussed. In fact, there is growing evidences supporting glutamate transporters are not static but extremely dynamic proteins, which can be found internalized in the intracellular space, and when in the membrane, display the ability to diffuse through glia surface (Pita-Almenar et al., 2012; Murphy-Royal et al., 2015), likely shaping the distribution of extracellular glutamate.

## Glutamate Transporters Impact Within the Synapse

Glutamate transporters and receptors have similar affinities for glutamate (Arriza et al., 1994), and taking into consideration that glutamate transport is not solely a mechanisms for shutting down neurotransmitter action, it should be noted that these can also be regarded as a diffusion sink capable of modifying synaptic responses on a millisecond time scale by sequestering glutamate at the binding sites within the transporter (Wadiche et al., 1995). Thus, glutamate transporters do play an important role in synaptic transmission, being crucial for maintaining optimal extracellular glutamate levels. AMPAR and NMDAR have a higher expression in the synapse, where glutamate transporters are unlikely to have any sort of competition, due to space constraints. Nevertheless, on account of its privileged localization, glutamate transporters may easily counteract the action of a fraction of extrasynaptic NMDAR in hippocampal synapse and mGlurRs, which are highly concentrated at the perisynaptic membrane of neurons (Baude et al., 1993). Accordingly, the activity of glutamate transporters can be modulated in order to regulate synaptic transmission. This is possible since glutamate transporters can act as diffusion sinks for glutamate and also as a consequence of their high dynamism in the membrane, allowing the adjustment of its activity in different stages of long-term potentiation (LTP). This modulation can be achieved by either exocytosis or endocytosis of transporters, membrane surface diffusion (Murphy-Royal et al., 2015; Al Awabdh et al., 2016) in co-cultures of neurons and astrocytes, brain slices and living mice. In the hippocampal neuropil, an area with a high-density of synapses, pharmacological blockade of glutamate transporters prolonged NMDAR-mediated Excitatory postsynaptic currents (EPSCs) in CA1 pyramidal neurons that had suffered high stimulation, but not in those subjected to low stimulation, suggesting glutamate transporters restrict glutamate spillover from neighboring synapses, and revealing that, in this region, independent synapses can collaborate with each other via glutamate transporter (Arnth-Jensen et al., 2002). Both LTP and long-term depression (LTD) induction depend on the activation of NMDAR and mGluR (Anwyl, 2009; Gladding et al., 2009; Lüscher and Malenka, 2012) and these receptors can be modulated by extracellular glutamate concentration, which is mediated by glutamate release in the synaptic cleft, glutamate diffusion plus glutamate uptake. In the CA1 region, neuronal glutamate transporters are able to control the level of NMDAR activation (Diamond, 2001) modulating neuronal excitability by regulating Kv2.1 channels (Mulholland et al., 2008). mGluR EPSCs are also potentiated by glutamate transporters inhibition in both cortex and hippocampus (Huang et al., 2004; Otis et al., 2004), constituting a possible candidate to suffer modulation by glutamate transporters. It appears that by competing with postsynaptic glutamate receptors, glutamate transporters mediate the level of activity of these receptors, making them relevant in synaptic plasticity.

### Glutamate Uptake in LTP

Long term potentiation is a form of synaptic plasticity where a persistent strengthening of synapses based on recent patterns of activity occurs (Bliss and Lomo, 1973), resulting in a long-lasting increase in signal transmission between neurons (for a deeper review see: Nicoll, 2017). Maintenance and modulation of LTP is usually associated with G-protein coupled receptor (GPCR) and/or protein phosphorylation, typically by a heightening of EPSCs (Betke et al., 2012). In the CA1 region, glutamate uptake is not homogenous throughout LTP and it is well established that LTP induction elicits glutamate uptake enhancement (Pita-Almenar et al., 2006, 2012) by increasing expression of glutamate transporters at the membrane. However, this modulation of uptake is regulated by distinct pathways in early-LTP and late-LTP. During early-LTP, glutamate uptake increase is insensitive to dihydrokainate (DHK), a selective GLT-1 inhibitor, being mostly secured by an enhanced expression of EAAC1 at the membrane level. There are no studies confirming an increase of glutamate transporters synthesis, just increase of uptake during LTP and transporter expression at the membrane level (Pita-Almenar et al., 2006). Conversely, late-LTP was DHK sensitive and required macromolecular synthesis mediated by phosphatase kinase C (PKC) (Pita-Almenar et al., 2006). Since PKC is a protein-related to Ca^2+^ signaling (Huang, 1989), this suggests that glutamate uptake modulation can be influenced by changes in intracellular Ca^2+^ concentration. This is noteworthy considering the majority of glutamate uptake is mediated by glial glutamate transporters, along with the fact that astrocytes show excitability by Ca^2+^ signaling (Araque et al., 1999; Perea and Araque, 2005; Sherwood et al., 2017). Actually, in astrocytic cultures, chelation of Ca^2+^ can prevent the modulation of glutamate and GABA transporters (Leonova et al., 2001; Matsuura et al., 2002; Cristovao-Ferreira et al., 2011; Jacob et al., 2014). LTP is impaired in hippocampal slices of GLT-1KO mice but this was overcome in the presence of low concentrations of NMDAR antagonists (Katagiri et al., 2001). This suggests that GLT-1 mediates NMDAR activity by controlling the levels of extracellular glutamate, and this is plausible explanation of how glutamate transporters modulate synaptic transmission, by regulating the activation of postsynaptic glutamate receptors via a control of the concentrations of glutamate present at the synaptic cleft.

As mentioned before, glutamate uptake mediated by glutamate transporters is an electrogenic process and, therefore, the concentrations of K^+^, Na^+^ and H^+^ directly influence glutamate uptake and, subsequently, synaptic plasticity. In glutamate transporters, the inward flow of a glutamate anion and three Na^+^ ions is simultaneously accompanied by the outflow of one K^+^ ion (Kanner, 2006). Therefore, glutamate uptake can be modulated by the extracellular concentration of both Na^+^ and K^+^ ions. Changes in synaptic plasticity by modulating concentrations of K^+^ has been correlated with glutamate transporters (Kucheryavykh et al., 2007; Mulholland et al., 2008). Knockout of astrocytic K^+^ channel impaired glutamate uptake and enhanced short term potentiation (Djukic et al., 2007). This can be due to the fact that increased extracellular K^+^ impairs the electrogenic process of the glutamate transporters, compromising normal uptake activity. EphA4, a receptor tyrosine kinase, reduces both GLT-1 and GLAST expression, leading to LTP impairment, which was rescued by pharmacological inhibition of glutamate transporters (Carmona et al., 2009). Surprisingly, we did not find any interactions of glutamate transporters with the sodium potassium pump (Na^+^/K^+^-ATPase), a ubiquitous membrane protein (Lees, 1991) at the electrophysiological level. However, there are some studies suggesting a possible correlation between these two proteins such as co-localization in the hippocampus (Rose et al., 2009). Ouabain, a specific antagonist of Na^+^/K^+^-ATPase, inhibits glutamate uptake in synaptosomes and also exhibits a bimodal effect by only inhibiting at high concentrations in cultures of astrocytes (Rose et al., 2009; Illarionava et al., 2014). In fetal human astrocytes, glutamate transporters activity can enhance Na^+^/K^+^-ATPase activity as well as cell surface expression (Gegelashvili et al., 2007), hinting on a possible interaction between these proteins. This can be interesting since Na^+^/K^+^-ATPase modulation has been correlated with changes in synaptic transmission (Scuri et al., 2007; Diaz et al., 2013).

**FIGURE 1:**
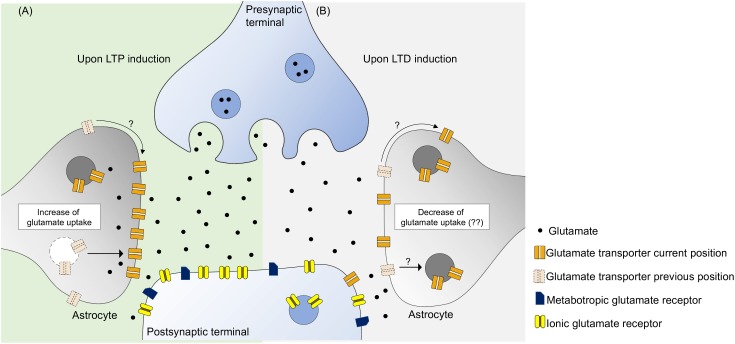
Glutamate transporters have the ability to monitor the concentration of synaptic glutamate, potentially controlling the activity of glutamate metabotropic and ionic receptors. **(A)** During LTP induction there is an increase of glutamate transport activity probably due to an increase of glutamate transporters near synaptic cleft. This can be due to a intracellular trafficking from the intracellular space to the membrane and surface diffusion to the synaptic region, leading to an optimal activation of postsynaptic receptors. **(B)** As for LTD induction, the specific role of glutamate uptake is yet to be revealed. It is know that increase of glutamate transporters decreases LTD, perhaps by not activating postsynaptic receptors to an optimal level. Furthermore, blockade of glutamate uptake enhances LTD suggesting that glutamate uptake may not be static during this phenomenon. We propose that there occurs some sort of glutamate uptake decrease either by removing glutamate transporters by internalization or by surface diffusion.

LTP induction and maintenance require optimal glutamate extracellular concentration (Katagiri et al., 2001), which is secured by glutamate transporters, mostly expressed in astrocytes, being glutamate transport a key factor for the induction and maintenance of hippocampal LTP. Hence, during LTP, there is an increase of glutamate uptake activity mainly in astrocytes (Figure 1A).

### Glutamate Uptake in LTD

LTD comprehends a form of synaptic plasticity where a weakening of synapses occurs by a reduction of the efficiency. This can be interpreted as an internalization of AMPA receptors in the postsynaptic membrane, triggered by synaptic activation of either NMDARs or mGluRs (Collingridge et al., 2010). The impact of glutamate uptake in LTD is not as well understood as in LTP, but some studies suggest a possible correlation between these two mechanisms. As glutamate transporters are not enzymes, there are no available agonists capable of enhancing its activity, making it difficult to “synthetically” elicit an increase in glutamate uptake. One way to do it is to treat living mice, brain slices or cultures with ceftriaxone, a beta-lactam antibiotic that enhances GLT-1 expression (Rothstein et al., 2005; Omrani et al., 2009; Bajrektarevic and Nistri, 2017). Chronic ceftriaxone treatment in Wistar rats increased GLT-1 expression, which produced an impairment in LTD in hippocampus mossy fibers CA3 (MF-CA3) synapses (Omrani et al., 2009), an effect reversed by the blockade of GLT-1 with DHK. This can be explained by a restraining of the level of activation of peri-synaptic mGluRs as a result of increased glutamate clearance by glutamate transporters, since, in these synapses, LTD is mGluR dependent (Yokoi et al., 1996), which, as above mentioned, is a receptor that can be modulated by glutamate uptake. Behavior can also affect the activity of glutamate transporters. It is known that stress enhances LTD and decreases LTP (Pan Wong et al., 2007). One study suggests that this stress-mediated LTD enhancement in the CA1 region of the hippocampus is done through the blockade of glutamate transporters, since both LTD enhancement and decreased uptake were reversed when animals had been previously treated with glucocorticoid receptor antagonist, RU38486, before stress induction (Yang et al., 2005). It is not exclusively in the hippocampus that glutamate transporters are relevant in LTD, existing connections in other brain regions, such as the amygdala (Tsvetkov et al., 2004) and the cerebellum (Brasnjo and Otis, 2001), where similar mechanisms can also occur.

To our knowledge, the role of glutamate transporters in hippocampal LTD, under physiological conditions, still remains elusive. Enhancement of glutamate uptake activity impairs LTD, however, this does not necessarily mean that uptake decreases in order to achieve LTD, although some alterations are expected. The fact that blockade of glutamate uptake enhanced LTD (Yang et al., 2005; Omrani et al., 2009), allied to increase produced the opposite effect, suggests that during LTD there can be a decrease of functional transporters in the membrane, leading to an optimal activation of glutamate receptors. Perhaps, in a similar way to what occurs with AMPA receptors, glutamate transporters are also internalized by cells and translocated to the plasma membrane upon an appropriate stimulus (Figure 1B). Further studies need to be made in order to grasp the role of glutamate transporters in LTD, in which transporters have not been subjected to any form of treatment.

## Conclusion

Glutamate clearance by high affinity transporters is essential for the maintenance of glutamate homeostasis, which requires a functional level of expression of glutamate transporters at the membrane level of both astrocytes and neurons. Glutamate uptake is not a static process and can be finely adjusted in accordance to synaptic needs. Glutamate transporters are able to control the level of activation of glutamate receptors by controlling the level of glutamate present at the synaptic level. These changes can be achieved either by an over or under expression of glutamate transporters, altered cellular trafficking or changes in the transporter conformation, all processes that affect the affinity of these transporters for glutamate. For LTP, glutamate uptake needs to be enhanced when compared to basal levels (Pita-Almenar et al., 2006, 2012). Noting that late LTP requires protein expression, an increase in glutamate transporters is not surprising at this point. Conversely, despite some reports suggest causality between glutamate uptake and LTD, this interaction remains poorly comprehended. An increase of glutamate uptake activity is not favorable for LTD maintenance (Omrani et al., 2009), while a stress-mediated inhibition of glutamate transporters enhances it (Yang et al., 2005). Nonetheless, we did not find what occurs to glutamate transporters throughout a “standard” LTD. In both LTP and LTD phenomena, the impact of glutamate transporters is seemingly achieved by a fine regulation of the activation of peri- and extrasynaptic NMDARs and mGluRs. Further studies are essential for a better comprehension of the importance of glutamate uptake for synaptic transmission and plasticity, namely by clarifying how these transporters modulate such physiological processes.

The study of transporter activity has proven to be particularly challenging, mostly on account of a lack of suitable methodologies. Techniques used for assessing receptor function are not applicable to transporters, and genetic approaches, as up- or down-regulating glutamate transport, although feasible, are not sensitive to variations in transporter activity, and thus, are unable to shed some light on the functional role of transporters during LTP or LTD. Blockade of glutamate transporters through the application of antagonists allows the inference of some of its putative functions in synaptic plasticity, however, it does not help in the clarification of the impact of enhanced glutamate transport. To our knowledge, the only current method is the application of ceftriaxone (Omrani et al., 2009), which acts by increasing GLT-1 expression levels and, concomitantly, by increasing glutamate uptake. In Murphy-Royal et al., 2015, GLT-1 surface diffusion was blocked by a cross-linking technique (Heine et al., 2008), which itself had an impact on synaptic transmission. Perhaps, the manipulation or the monitoring of this diffusion of transporters at an *in situ* or *in vivo* model could constitute a suitable approach for better comprehending how exactly do glutamate transporter influence synaptic signaling and plasticity. Additionally, glutamate imaging could also allow a more direct observation of the behavior of glutamate transporters in seconds, or even millisecond, timescale (Dulla et al., 2008; Okubo et al., 2010), and it can constitute a means for understanding how glutamate transporters impact glutamate dynamics (Hefendehl et al., 2016; Pinky et al., 2018). Whole cell patch clamp constitutes a powerful tool for measuring glutamate uptake in astrocytes (Bergles and Jahr, 1997), and dual-patch recordings could emerge as a plausible method for simultaneously recording glutamate uptake and neuronal excitability, and ultimately, correlate these two events.

In sum, sufficient evidence indicates the critical participation of glutamate transporters in synaptic transmission and synaptic plasticity in the hippocampus and other areas, and additional studies are required in order to better comprehend the mechanisms by which glutamate transporters and, subsequently, glutamate uptake impacts synaptic transmission and plasticity.

## Author Contributions

All authors listed have made a substantial, direct and intellectual contribution to the work, and approved it for publication.

## Conflict of Interest Statement

The authors declare that the research was conducted in the absence of any commercial or financial relationships that could be construed as a potential conflict of interest.
